# The Cholera Conference

**Published:** 1866-02

**Authors:** 


### The Cholera Conference.

The French Government has proposed to the different nations of Europe to send delegates of medical men to a Convention to be held at Constantinople, to discuss in the fullest manner all the questions concerning the origin and cause of Asiatic Cholera, with a view to the establishment of such sanitary and restrictive measures as shall seem the most likely to limit its ravages now, and check them at the outset hereafter.

In this Convention the United States have been invited to take a part; and a few days since the President sent to Congress a message transmitting the correspondence of the Secretary of State with the French Minister, together with other papers relating to the proposed international Convention. The Secretary of State promises to give the subject his attentive consideration, and on the 21st of last November asked the opinion of the Surgeon-General of the United States, for any suggestions he might be disposed to make in the premises. In reply, the Surgeon-General proposes that he be empowered to designate two of the Surgeons of the Medical Staff of the United States Army as members of such a commission. The Government has also received assurances of the cordial co-operation of the Turkish Government in the proposed conference. Lallah Effendi, the chief physician of the Imperial Court, and Dr. Bartholette, Counsel of Health, being nominated as representatives. A cordial welcome to the delegates is promised by the Court.

